# Small Molecules for the Treatment of Long-COVID-Related Vascular Damage and Abnormal Blood Clotting: A Patent-Based Appraisal

**DOI:** 10.3390/v16030450

**Published:** 2024-03-14

**Authors:** Francesco Samarelli, Giovanni Graziano, Nicola Gambacorta, Elisabetta Anna Graps, Francesco Leonetti, Orazio Nicolotti, Cosimo Damiano Altomare

**Affiliations:** 1 Department of Pharmacy—Pharmaceutical Sciences, University of Bari Aldo Moro, I-70125 Bari, Italy; francesco.samarelli@uniba.it (F.S.); giovanni.graziano@uniba.it (G.G.); nicola.gambacorta1@uniba.it (N.G.); francesco.leonetti@uniba.it (F.L.); orazio.nicolotti@uniba.it (O.N.); 2 ARESS Puglia—Agenzia Regionale Strategica per la Salute ed il Sociale, I-70121 Bari, Italy; e.graps@aress.regione.puglia.it

**Keywords:** long COVID, vascular issues, ramatroban, caspase inhibitors, procyanidins

## Abstract

People affected by COVID-19 are exposed to, among others, abnormal clotting and endothelial dysfunction, which may result in deep vein thrombosis, cerebrovascular disorders, and ischemic and non-ischemic heart diseases, to mention a few. Treatments for COVID-19 include antiplatelet (e.g., aspirin, clopidogrel) and anticoagulant agents, but their impact on morbidity and mortality has not been proven. In addition, due to viremia-associated interconnected prothrombotic and proinflammatory events, anti-inflammatory drugs have also been investigated for their ability to mitigate against immune dysregulation due to the cytokine storm. By retrieving patent literature published in the last two years, small molecules patented for long-COVID-related blood clotting and hematological complications are herein examined, along with supporting evidence from preclinical and clinical studies. An overview of the main features and therapeutic potentials of small molecules is provided for the thromboxane receptor antagonist ramatroban, the pan-caspase inhibitor emricasan, and the sodium–hydrogen antiporter 1 (NHE-1) inhibitor rimeporide, as well as natural polyphenolic compounds.

## 1. Introduction

The most observed symptom of COVID-19, the pandemic infection caused by the Severe Acute Respiratory Syndrome Coronavirus 2 (SARS-CoV-2) [1,2], is pneumonia, which is associated with dry cough, dyspnea, and fever. Other symptoms may include gastrointestinal disorders, leucopenia, tiredness, and/or loss of appetite. In the most severe cases, respiratory failure, which requires treatment in intensive care units using mechanical ventilation, can occur.

COVID-19 severity was associated with hyperinflammation, which is characterized by activation of the innate immune response, including the so-called cytokine storm—an excessive or uncontrolled release of proinflammatory cytokines [2]. For this reason, anti-inflammatory interventions have been thought to block persistent inflammation, also due to the cytokine storm [3,4,5,6,7,8]. Moreover, an elevated diffuse inflammatory cytokine profile was associated with post-acute sequelae of SARS-CoV-2 (PASC), commonly known as long COVID. It has been shown that long COVID can involve other pathways different from the cytokine storm in acute COVID-19, for example, an upregulated neutrophil-associated immune signature characterized by an increase in chemokines and proteases, as well as markers of neutrophil extracellular traps in blood [9].

Regardless of the viral status, long COVID refers to the existence of numerous symptoms even weeks or months after contracting SARS-CoV-2 infection. Depending on the length of symptoms, long-term COVID is characterized by two stages: (i) post-acute and (ii) chronic COVID, whose symptoms last from three to twelve weeks and longer than twelve weeks, respectively [10]. Overall, long COVID is difficult to tackle not only in terms of therapies but also for properly assessing the transmission potential. While respiratory transmission is the main method of spreading SARS-CoV-2, the dynamics of long COVID onset reveal a complex situation determined by multiple factors. As a matter of fact, the latter is a complex infection based on hidden interactions between the host immune system, viral persistence, and other variables [11,12]. Long COVID may be linked to persistent viral reservoirs or fragments in other tissues, raising concerns about possible transmission through physiological fluids or other pathways irrespective of the respiratory system. Distinct features of long COVID regarding immune profiling and pathogenic mechanisms underlying the chronic disease have been recently updated [13,14].

The knowledge of long COVID transmission is further complicated by the variety and heterogeneity of its manifestations. Furthermore, long COVID is difficult to diagnose. The clinical recovery time varies depending on the severity of the sickness, and the associated comorbidities make the determination of a cut-off point for diagnosis difficult [15]. In this respect, the persistent and often unpredictable nature of long COVID symptoms represents a unique set of challenges for patients and healthcare providers. In fact, although respiratory conditions are frequently present, neurological, gastrointestinal, and cardiovascular issues can also arise [16].

Although the mechanisms of COVID-19-associated coagulopathy (CAC) have not been fully elucidated, CAC showed unique features [17]. Indeed, minimal changes in platelet counts, normal thrombin times, and increasing levels of D-dimer and fibrinogen were observed, and platelet activation was a main factor leading to inflammation and thrombogenesis, even though the effects of antiplatelet drugs have not been fully proven [18,19]. Evidence of how high levels of interleukin-6 (IL-6) stimulate megakaryocytopoiesis in the bone marrow and abnormal lung immunothrombosis in severe COVID-19 patients has been proven in postmortem and biopsy examinations [20]. The literature shows the importance of caspase activation in acute COVID-19, proving the effect of defibrotide, a mixture of single-stranded oligonucleotides extracted from the intestinal mucosa of pigs, in mitigating caspase 8 activation in microvascular endothelial cell (MVEC), cell injuries, and related vasculopathies [21], suggesting a potential benefit of this biological drug when used early in the disease course.

Herein, we focus on interconnected inflammation, vascular endothelial dysfunction, and abnormal blood clotting, which are among the main adverse outcomes of long COVID. Neuroinflammation, damage to cerebral circulation, and endothelial dysfunction also underlie post-COVID neurological disorders and cognitive impairments. A recent investigation of factors associated with viral persistence, chronic inflammation, and hypercoagulability and neurocognitive issues proposed a mechanism that highlights the impact of serotonin reduction in post-acute SARS-CoV-2 infection [22]. Proinflammatory and prothrombotic disorders are treated with drugs ranging from antiplatelet agents such as aspirin, dipyridamole, and thienopyridines, to anticoagulants such as heparin and direct thrombin/factor Xa inhibitors [23]; however, their effects in COVID-19 patients have not yet been definitively proven.

In this study, we wanted to review alternative therapies under evaluation and/or repositioned drugs for treating vascular syndromes associated with long COVID by retrieving patents submitted in the last three years and clinical trial information from the main respective websites [24,25] along with the related preclinical literature.

## 2. Vascular Disease and Blood Clotting in Long COVID

Long COVID demonstrates a concerning pattern of cardiovascular abnormalities that extend beyond the acute infection. Patients report persistent symptoms such as chest discomfort and palpitations, and viremia-associated effects on the heart and vasculature. One major source of concern is the possibility of direct viral invasion of cardiac tissues, resulting in myocarditis. This inflammatory condition, which has been observed both in the acute phase and as a lingering effect of chronic COVID, raises worries about long-term heart muscle damage and subsequent cardiovascular issues. In addition, endothelial dysfunction, which is a critical factor in cardiovascular health, appears as a central feature during long COVID. Because of the virus’ potential to interfere with the delicate balance in the endothelium, decreased vascular function may be the basis for atherosclerosis and increased vulnerability to cardiovascular events [26,27].

SARS-CoV-2 affects all organs, including the circulatory system. It had been hypothesized that persistent inflammation may aggravate cardiovascular risks, but very recent findings proved that it is complement activation, rather than the persistent inflammation, that triggers thrombosis associated with long COVID disease [28]. Chronic inflammation contributes to endothelial dysfunction, vasculitis and vasculopathy [29,30], plaque formation, and an increased risk of thrombotic events, highlighting the complex interplay between a chronic inflammatory state and cardiovascular issues [31,32,33]. Recognizing cardiovascular complications as a consequence of chronic COVID has significant public health implications. Healthcare providers must remain vigilant in monitoring and managing the cardiovascular health of people affected by long COVID, recognizing the possible long-term burden on healthcare systems as well as the well-being of those dealing with the long-term effects of SARS-CoV-2 infection. The use of antiplatelet and anticoagulant agents against COVID-19 has been investigated in the last few years [34,35,36]; however, no significant impact of the antiplatelet drugs on its morbidity and mortality has been proven [19], whereas heparins can be helpful in non-critically ill COVID-19 patients.

The molecular biology underlying the infection mechanisms of SARS-CoV-2 reveals that among the macromolecules crucial for the entry of the virus into host cells are at least two proteins linked to the cardiovascular system, namely the angiotensin-converting enzyme 2 (ACE2) and transmembrane protease, serine 2 (TMPRSS2). ACE2 converts angiotensin I to angiotensin II, which causes increased vessel contraction when it binds to AT1 receptors, ultimately resulting in an increase in blood pressure. ACE2 is also the primary cell surface receptor recognized by the Spike protein of SARS-CoV-2 [37]. The use of ACE2 inhibitors (e.g., captopril, enalapril, and lisinopril) in COVID-19 therapy was initially hypothesized to reduce the risk of death in infected patients, but it has been shown that the use of ACE inhibitors is not associated with an increase or reduction in the risk of all-cause mortality compared, for instance, to the use of calcium channel blockers [38].

**Figure 1:**
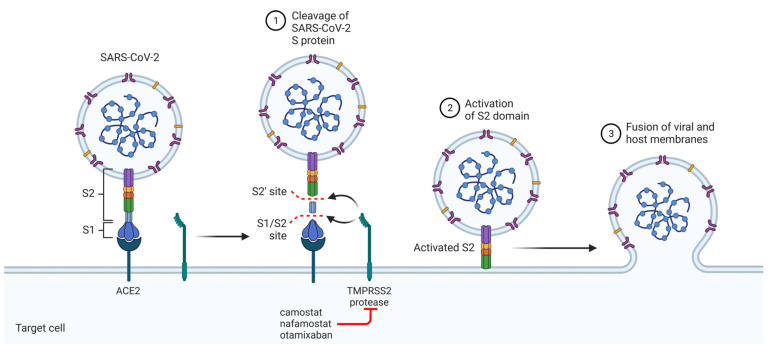
Representation of the mechanism of entry of the virus into host cells. Angiotensin-converting enzyme 2 (ACE2) is responsible for recognizing the Spike protein. Transmembrane protease, serine 2 (TMPRSS2) catalyzes the proteolytic cleavage that allows fusion between the virus membrane and the host cell membrane. Camostat, nafamostat, and otamixaban inhibit TMPRSS2.

TMPRSS2 is a serine protease belonging to the S1A family. It has a high degree of homology with other serine proteases of the same S1A family involved in coagulation processes, such as coagulation factor Xa (80% homology at the active site) [39]. TMPRSS2 colocalizes with ACE2 on the cell membrane in the aerodigestive tract, liver, kidneys, and sex organs, and is essential for viral spread and pathogenesis in the infected host. TMPRSS2 cleaves the Spike protein at two distinct protease cleavage sites, one located at the interface of the Spike protein subunits, termed S1/S2, and another within the S2 subunit, termed S2. These cleavages enable conformational changes that favor fusion between the viral and host cell membranes (Figure 1).

**Figure 2:**
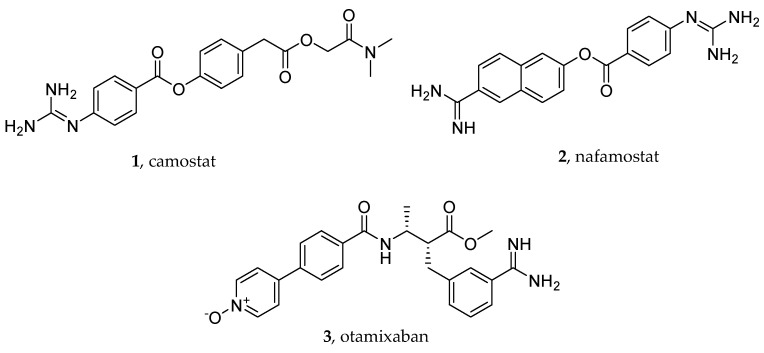
Chemical structures of some protease inhibitors, including direct oral anticoagulants (DOACs), acting also as covalent (**1** and **2**) and reversible (**3**) inhibitors of TMPRSS2, the serine protease catalyzing the activation of the Spike protein and ultimately SARS-CoV-2 entry into the host target cell.

These observations led to the evaluation of the repurposing of serine protease inhibitors, including some direct oral anticoagulant (DOAC) drugs such as nafamostat, camostat, and otamixaban (Figure 2, structures **1**–**3**), for COVID-19 therapy [35], with the final aim being to identify molecules capable of acting both at the level of virus entry (inhibiting TMPRSS2, hence protease activation of the Spike protein) and the downstream thrombotic complications [36].

**Figure 3:**
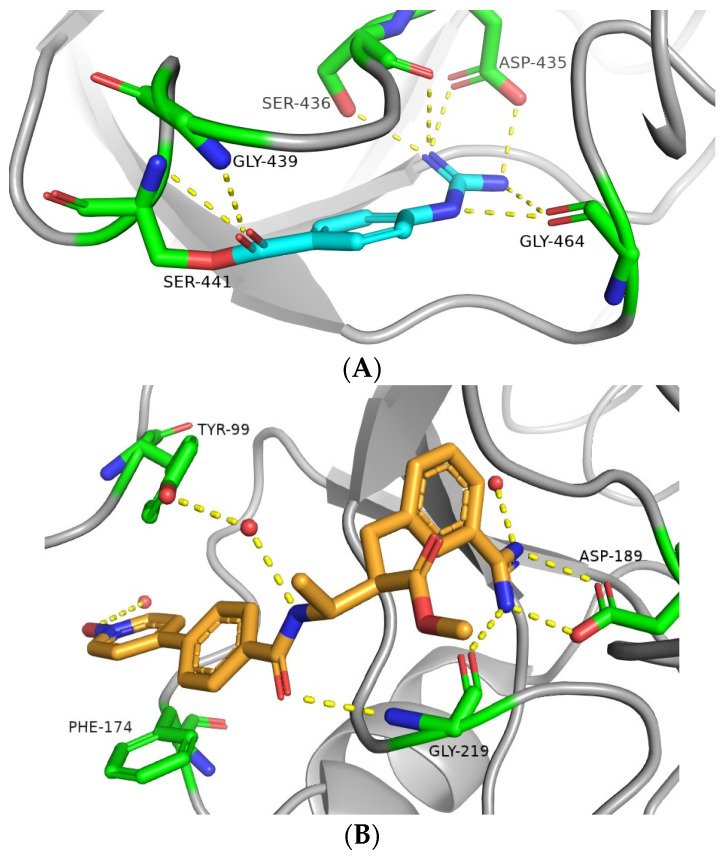
Crystal structures of (**A**) human TMPRSS2 with the catalytic Ser-441 acylated with 4-guanidinobenzoyl moiety of the covalent inhibitor nafamostat (PDB code: 7MEQ) [41]; (**B**) human blood coagulation factor Xa complexed with the reversible inhibitor otamixaban (PDB code: 1KSN) [42]. The ligands and key residues are rendered as sticks. C atoms of the ligands are colored cyan (nafamostat, A) and orange (otamixaban, B); C atoms of the contacting residues are colored green, whereas N and O are colored blue and red, respectively; H atoms are omitted for clarity. Water molecules are represented by small red spheres and H-bond interactions are shown as yellow dashed lines.

Some DOACs were not only shown to decrease the risk of death in infected patients [40] but also proved to be effective at reducing SARS-CoV-2 infection through the inhibition of TMPRSS2. For instance, camostat (**1**) and nafamostat (**2**), two guanidine/amidine-containing anticoagulant agents, have been identified as covalent TMPRSS2 inhibitors (Figure 3A) [41]. Moreover, otamixaban (**3**), a phase 3 clinical trial candidate originally developed as a selective reversible inhibitor of the blood coagulation factor Xa (Figure 3B), was found to inhibit SARS-CoV-2 viral entry. The IC_50_ values determined in enzyme inhibition assays with TMPRSS2 and in virus-infected Calu-3 cells fell within the μM range (2.55 and 18.7 μM, respectively), and the experiments on precision cut human lung slices showed that, in lung tissue, otamixaban was as potent as camostat, and the combination of otamixaban with low doses of nafamostat or camostat significantly enhanced its effect in a synergistic manner [35]. Subsequently, Hempel T. et al. patented a pharmaceutical composition consisting of otamixaban and nafamostat or camostat to treat or prevent SARS-CoV-2-related diseases.

**Figure 4:**
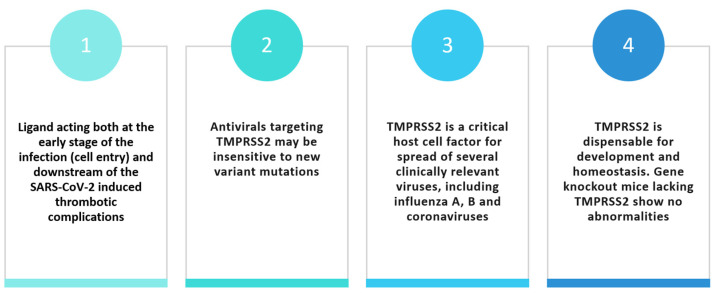
Potential advantages of TMPRSS2 inhibitors for the treatment of COVID-19.

The advantages of this approach would not be limited to the dual action on virus entry (through TMPRSS2 inhibition) and downstream thrombotic complications (fXa inhibition) (Figure 4). Antivirals directed toward host proteins may be insensitive to new variant mutations. In addition, TMPRSS2 is a critical host cell factor for spreading several clinically relevant viruses, including influenza A and B and coronaviruses. TMPRSS2 is dispensable for development and homeostasis, and gene-knockout mice lacking TMPRSS2 show no abnormalities [43].

In the context of COVID-related cardiovascular issues, we identified recently patented compounds used for the treatment of cardiovascular complications associated with long-term COVID, namely natural or nature-inspired polyphenolic compounds, ramatroban, a dual antagonist of PGD2/DPr2 and thromboxane A2/TPr receptors [44,45], procyanidins [46,47], NHE-1 inhibitors [48], and caspase inhibitors [49]. Table 1 summarizes the molecules discussed hereafter, with their target(s)/mechanism(s) of action, the phase of clinical trials or preclinical investigation. Based on this wealth of information, this survey focuses on the evaluation of the therapeutic potential of these compounds in the treatment of vascular diseases associated with long COVID.

## 3. Thromboxane A2 Receptor Antagonists

During SARS-CoV-2 infection, cyclooxygenase enzymes (COXs), especially COX-2, are upregulated and increase the expression and release of lipid mediators [50]. A targeted lipidomic analysis of bronchoalveolar lavages (BALs) using tandem mass spectrometry performed on 25 healthy controls and 33 COVID-19 patients requiring mechanical ventilation revealed that in addition to the well-known cytokine storm [51], a lipid mediator storm also occurs in infected patients, with a predominance of prostaglandins and thromboxane. The most increased COX metabolite was TXB2 (the stable metabolite of TXA2) >> PGE2~12-HHTrE > PGD2 [52].

PGD2 interacts with prostanoid receptor DP2, causing bronchoconstriction and promoting inflammation. PGD2/DP2 receptor signaling suppresses interferon-λ expression, whereas DP2 receptor antagonism stimulates interferon-λ expression and suppresses viral replication. On the other hand, TXA2 has prothrombotic properties. It modulates the functions of platelets and endothelial cells in a paracrine manner via the thromboxane receptors (TPrs) and promotes the activation of the coagulation cascade [44].

**Figure 5:**
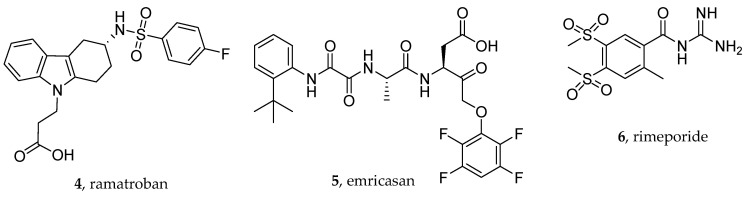
Chemical structures of the thromboxane receptor antagonist ramatroban (**4**), the pan-caspase inhibitor emricasan (**5**), and the sodium–hydrogen antiporter 1 (NHE-1) inhibitor rimeporide (**6**).

**Figure 6:**
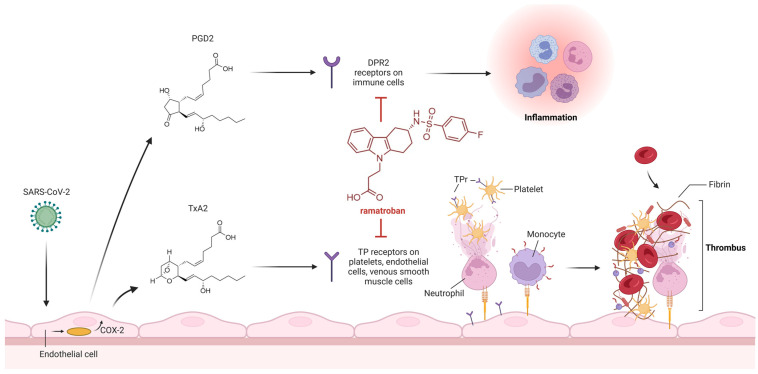
Schematic representation of the mechanism of action of ramatroban, a dual prostaglandin D2 receptor (DPr2) and thromboxane A2 receptor (TPr) antagonist in clinical trials for thromboinflammatory dysregulation in acute and long COVID-19.

**Figure 7:**
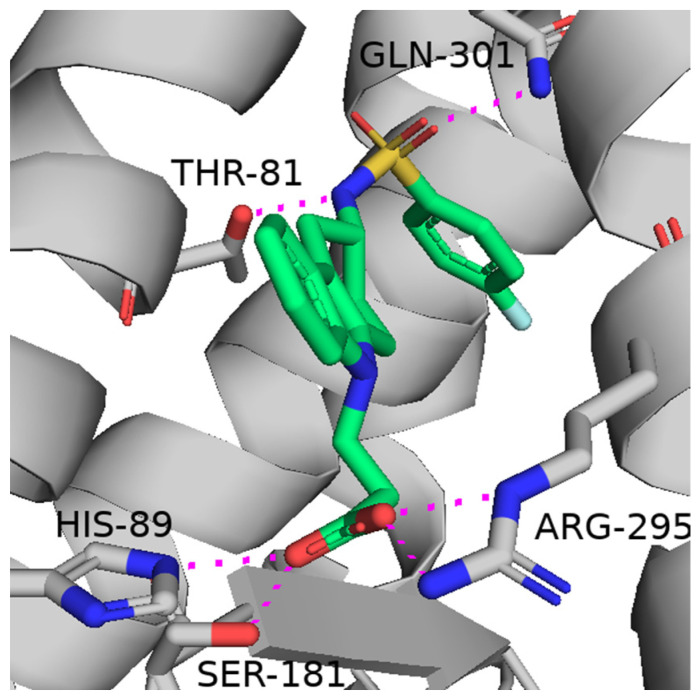
Binding pose of ramatroban in the human thromboxane A2 receptor (PDB code: 6IIU). The ligand and key residues are rendered as sticks and C atoms colored pale green and grey, respectively, whereas N, O and S are colored blue, red and yellow, respectively; Hydrogens are omitted for clarity. Hydrogen bonds are shown as magenta dotted lines.

As claimed by patent no. US11583517B2, ramatroban (Figure 5), a dual DP2/TrP antagonist, blocking the deleterious effects of both PGD2 and TXA2 (Figure 5) may be beneficial against COVID-19, as recently suggested by Gupta A. et al. [45] (Figure 6). Ramatroban (**4**) binds to thromboxane A2 receptors (TPrs) in human platelets (Figure 7) with a *K*_i_ value of 10–13 nM, and was found to antagonize the PGD2 receptor, significantly inhibiting the binding of [^3^H]PGD2 with an IC_50_ value of 100 nM [53,54]. It has a well-established safety profile and has been used orally as a treatment for allergic rhinitis in Japan since 2000 [55]. Ramatroban provided rapid relief from symptoms in four patients with severe COVID-19 [50]. To further confirm these data, a randomized, double-blind, placebo-controlled, parallel-design, multicenter, adaptive phase-2/phase-3 study to evaluate the efficacy and safety of ramatroban along with the standard of care in subjects hospitalized with SARS-CoV-2 infection is ongoing (NCT05706454).

The study is currently recruiting participants who will be randomized in a 1:1 ratio to two treatment groups. Group I will be treated with ramatroban (75 mg tablet) and given the standard care; group II will be treated with a placebo and given the standard care.

The aim of the study is to assess the efficacy and safety of ramatroban in subjects hospitalized with COVID-19 and to evaluate its long-term effects on PASC. In particular, the study aims to (i) examine the lipid mediators, specifically TXA2, PGD2, F2-isoprostane, and/or their metabolites in convalescent subjects after treatment, and (ii) assess the efficacy of ramatroban administered during acute illness in preventing or mitigating subsequent development of long COVID. The results of the ongoing clinical trials are needed to validate the hypotheses of what appears to be a promising therapy for the treatment of COVID-19 and long-COVID-related vascular disorders.

## 4. Caspase Inhibitors

Caspases are a family of intracellular cysteine protease enzymes playing essential roles in programmed cell death. Since their key role in apoptosis was identified in 1993, twelve caspases, which carry out a variety of cellular functions, have been discovered in humans [56,57]. Caspases play other roles in programmed cell death, including pyroptosis (a highly inflammatory form of lytic programmed cell death), necroptosis (a programmed form of necrosis or inflammatory cell death), and PANoptosis (an inflammatory cell death pathway). Cell death mechanisms are crucial for maintaining an optimal environment for proper cell function. However, during SARS-CoV-2 infection or viral infection in general [58], the dysregulation of these systems contributes to disease pathogenic mechanisms. SARS-CoV-2 can induce host cell death via five types of regulated cell deaths, i.e., apoptosis, necroptosis, pyroptosis, autophagy, and PANoptosis [59].

Targeting the innate immune system pathways may be a good therapeutic option, as demonstrated for example by monoclonal antibodies such as tocilizumab and sarilumab, two IL-6 receptor blockers, which in fact proved to be effective at reducing the severity of COVID-19 outcomes [60,61]. For example, the caspase-8 inhibitor Z-IETD-FMK subdued SARS-CoV-2-induced BID cleavage and caspase-3 activation [62]. In another study, Plassmeyer et. al. [49] investigated caspase-1 activity in SARS-CoV-2 infection, including in red blood cells, given the significance of COVID-19-associated coagulopathies [63,64]. Transcriptional states of multiple caspases and expression of active caspase-1 in blood cells from COVID-19 patients in acute and convalescent stages of the disease were evaluated. Non-COVID-19 subjects presenting comorbidity conditions served as controls. In addition, suppression of caspase-1 activity was evaluated using caspase inhibitors; whole blood samples from patients were incubated with oral pan-caspase inhibitor emricasan (EMR) or ICE/caspase-inhibitor 1 selective orally active VX765 [65,66]. The pan-caspase inhibitor emricasan (**5**, Figure 5) was shown to attenuate caspase-1 hyperactivity in CD4+ T cells from COVID-19 patients ex vivo.

EMR (**5**) not only inhibits caspase-1 (and other caspases) but may also inhibit the activity of the main SARS-CoV-2 protease as well as SARS-CoV-2 binding to the ACE-2 receptor. Such multi-target activity may prove useful in the treatment of infections caused by positive-strand RNA viruses besides SARS-CoV-2 [67,68].

A tolerability study was conducted by Raavi Gupta (NCT04803227 [69]) in 50 symptomatic patients with mild COVID-19 by administering EMR at 25 mg BID dosing for 14 days, with a 1:1 active:placebo ratio of mild COVID-19 patients receiving standard care therapy [69]. Although EMR has been extensively studied in humans in phase-1 and phase-2 studies, where it has shown excellent safety and tolerance profiles, it had not been used in the COVID-19 setting, therefore necessitating an initial safety and tolerability study [40,70,71]. The study was terminated because of unforeseeable difficulties in recruiting patients. However, no serious adverse events were reported. As described in patent no. WO 2023018991A1 published by the World Intellectual Property Organization in February 2023, EMR can be used as a therapy against COVID-19 characterized by or associated with microthrombosis.

## 5. Polyphenols

Endothelial dysfunction, defined as poor blood vessel lining function, is emerging as a possible risk factor for long COVID [72,73]. This dysfunction is linked to the inflammatory response induced by SARS-CoV-2, which results in chronic inflammation, vascular damage, and blood hypercoagulability. These effects could be amplified by direct viral influence on blood vessels. Moreover, inappropriate therapy and the connection of bacterial infection, with an increase in inflammatory mediators, may result in several chronic diseases that require longer recovery periods. COVID-19 inflammatory cytokines present after the acute phase are therapeutic targets for inflammatory illnesses [74]. Proanthocyanidins, a group of flavonoids found in various plant-based foods, may be utilized to decrease, or even neutralize, the symptoms of the post-COVID-19 inflammatory state. These are polyphenolic antioxidants able to fight cell-damaging free radicals, providing cardiovascular protection and anti-inflammatory activity.

**Figure 8:**
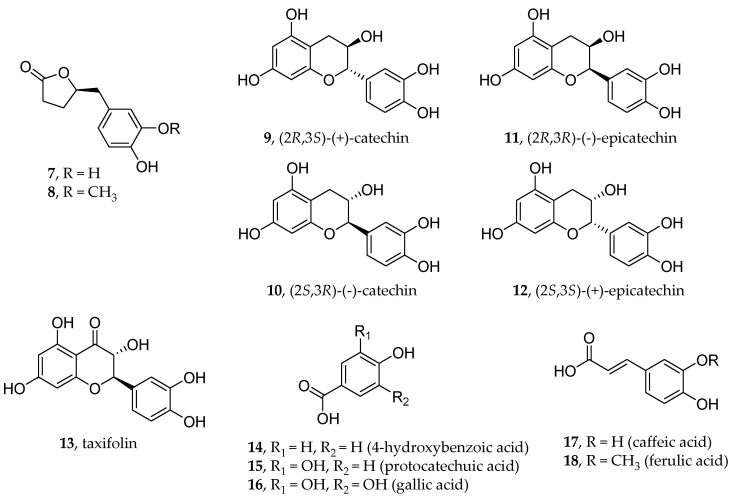
Structures of flavonoid and non-flavonoid polyphenols as potent antioxidants contained in the proanthocyanidin-rich extract Pycnogenol^®^.

Several studies [75,76,77,78] and a phase 3 clinical trial (NCT05890534) proved that Pycnogenol^®^, a proanthocyanidin-rich extract from the bark of French maritime pine, enhances endothelial function by stimulating endothelial nitric oxide synthase (eNOS), which induces the production of nitric oxide (NO), resulting in an increase in vessel lumens and appropriate tissue perfusion. Horphag Research (Limassol, Cyprus) has patented Pycnogenol^®^ for use in the treatment of endothelial dysfunction triggered by COVID-19. The extract contains 70–75% w/w proanthocyanidins as sources of polyphenolic compounds such as δ-(3,4-dihydroxyphenyl)-γ-valerolactone (**7**) and its 3-methoxy analog (**8**), diastereomeric catechins and epicatechins (**9**–**12**), taxifolin (**13**), 4-hydroxybenzoic acid (**8**), protocatechuic acid (**14**), gallic acid (**15**), caffeic acid (**16**), and ferulic acid (**17**) (Figure 8), which can be used as therapies against COVID-19-related endothelial inflammation and/or endothelial systemic dysfunction.

Moreover, the patent reports the employment of three daily doses of 50 mg of Pycnogenol^®^ as supplement therapy in subjects suffering from COVID-19. After three weeks of treatment, the subjects achieved an improvement in both endothelial function and microcirculation, as well as a decrease in the inflammatory marker IL-6.

## 6. NHE-1 Inhibitors

The Sodium–Hydrogen Exchanger 1 (NHE-1) protein is a membrane transport protein playing a crucial role in maintaining intracellular pH homeostasis, especially in cardiac and renal cells. NHE-1 inhibition can have a variety of therapeutic consequences, making it an exciting field of research and drug development [79]. The major function of NHE-1 is to exchange internal sodium ions for extracellular hydrogen ions, which aids in cell pH regulation. This mechanism is critical in cardiac myocytes because it helps to maintain adequate myocardial contractility and electrical activity. Furthermore, NHE-1 participates in acid–base balance and sodium reabsorption in renal tubular cells. NHE-1 activity dysregulation can result in a variety of clinical diseases, including cardiac hypertrophy, heart failure, and renal problems.

NHE-1 inhibitors have shown promise as prospective treatments for a variety of cardiovascular diseases [80]. Given their inhibitory activity, these compounds can limit the entry of sodium ions into cardiac cells, preventing excess sodium, thus reducing the risk of cardiac hypertrophy and heart failure, both of which are often linked to improper calcium management and increased burden on the heart [81]. NHE-1 inhibitors may also have anti-arrhythmic properties, making them useful in the treatment of cardiac arrhythmias. Furthermore, by decreasing salt reabsorption in the renal tubules, these inhibitors can aid in blood pressure regulation and management of diseases such as hypertension and edema. They may also be useful in the treatment of metabolic acidosis.

**Figure 9:**
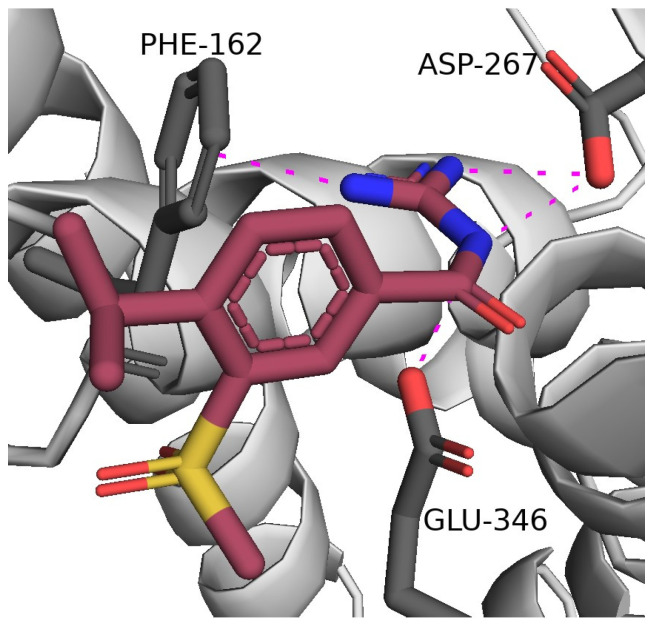
Binding pose of cariporide in the human NHE-1CHP1 complex (PDB code: 7DSX). The ligand and key residues are rendered as sticks and C atoms colored raspberry red and grey, respectively, whereas N and O are colored blue and red, respectively; Hydrogens are omitted for clarity. Hydrogen bonds and polar interactions are shown as magenta dotted lines.

In this scenario, given the importance of NHE-1 inhibitors in the treatment of cardiovascular diseases, their usage has been proposed and patented by Florence Porte-Thomé (Geneve, CHF) to treat both acute and long-term cardiovascular complications of SARS-CoV-2 infection such as heart failure, arrythmia, and cardiac ischemia [48]. More specifically, rimeporide (Figure 5), a well-established NHE-1 inhibitor [82], may exert beneficial effects on myocardial fibrosis or post-COVID pulmonary fibrosis. Figure 9 highlights key interactions of cariporide (an analog of rimeporide), which bears a 4-isopropyl group instead of the 4-methylsulfonyl group in rimeporide.

## 7. Conclusions

Long-term effects of SARS-CoV-2 infection include, among the severe adverse outcomes, cardiovascular complications encompassing endothelial dysfunction, inflammation, myocarditis, abnormal blood clotting, coagulopathies, thrombosis, and myocardial infarction. Long COVID is therefore a multi-organ disease, as the vascular and coagulation abnormalities affect every organ system. Although the interconnection between the proinflammatory and prothrombotic processes in the context of COVID-19 and long COVID is not yet fully understood, clinical practice normally uses combined anti-inflammatory and anticoagulant therapy.

Focusing on reviewing the recent patent literature and related preclinical supporting evidence, as well as some ongoing clinical trials, antioxidant polyphenol-rich extracts, the synthetic thromboxane inhibitor ramatroban, and other synthetic small-molecule drugs like emricasan (a pan-caspase inhibitor) and rimeporide (an NHE-1 inhibitor) emerged as candidates for the treatment of endothelial dysfunction, anomalous blood clotting, and oxidative stress underlying COVID-related cardiovascular pathologies. The results of ongoing and future clinical trials are expected to validate the use of these small molecules, even in combination therapy, as efficacious and safe agents for improving outcomes for people affected by cardiovascular syndromes associated with SARS-CoV-2 and similar virus infections, such as those caused by viruses belonging to the same SARS-CoV and MERS-CoV family.

## Figures and Tables

**Table 1:** Summary of small molecules studied as potential treatments of long-COVID-associated vasculopathies and blood clotting abnormalities, along with information on the mechanisms of action and the state of clinical investigation.

Molecules	Target(s)/Mechanism(s) of Action ^a^	Phase of Clinical or Preclinical Investigation
**Ramatroban**	DPr2 and TPr receptor antagonist	Phase 2/3
**Emricasan**	Pan-caspase inhibitor	Phase 1 (terminated)
**Pycnogenol^®^**	Antioxidant, eNOS	Phase 3
**Rimeporide**	NHE-1 inhibitors	Preclinical investigation

^a^ DPr2: Prostaglandin D2 receptor; TPr: Thromboxane receptor; eNOS: Endothelial nitric oxide synthase; NHE-1: Sodium–Hydrogen Exchanger 1.

## Data Availability

No new data were created.
